# Cognitive Processes and Legal Capacity in Patients With Bipolar Disorder: A Brief Research Report

**DOI:** 10.3389/fpsyg.2022.867750

**Published:** 2022-06-30

**Authors:** Fabiana Saffi, Cristiana C. A. Rocca, Edgar Toschi-Dias, Ricardo S. S. Durães, Antonio P. Serafim

**Affiliations:** ^1^Department of Psychology and Neuropsychology, Institute of Psychiatry, University of São Paulo, São Paulo, Brazil; ^2^Heart Institute, School of Medicine, University of São Paulo, São Paulo, Brazil; ^3^Health Psychology Program, Methodist University of São Paulo, São Paulo, Brazil

**Keywords:** bipolar disorder, cognitive deficit, euthymic bipolar patients, executive functioning, legal capacity

## Abstract

The current study verified the association between cognitive process such as attention, executive functioning, and legal capacity in patients with bipolar disorder (BD). The sample consisted of 72 participants, assorted to episodic patients (*n* = 21), euthymic patients (*n* = 22), and healthy controls (HCs) (*n* = 29). We used the following neuropsychological measures: subtests of the Wechsler Abbreviated Intelligence Scale (WASI): vocabulary and matrix reasoning; Continuous Performance Test (CPT); Five Digit Test (FDT); and Rey–Osterrieth Complex Figure (ROCF). Euthymic patients expressed slower processing speed (FDT) compared to HC. They tended to make more errors with slightly worse discrimination, suggesting more impulsiveness (CPT, *p* < 0.01). On the contrary, episodic patients showed worse discrimination, committed more omissions, were more inconsistent with regard to response speed (CPT-3, *p* < 0.01), showed more difficulties in organizing their actions (ROCF: copy, *p* = 0.03), and were more rigid (FDT: flexibility, *p* = 0.03). The results suggest that bipolar patients in episode express more cognitive impairments that can compromise the quality of legal capacity. These results highlight the need for more protective support for episodic BD patients regarding legal capacity.

## Introduction

There is growing evidence for the impact of mental disorders on the global functioning of people. These disorders are among the top 10 factors of disability among the population aged 15–44 years old (Merikangas et al., 2009). This scope includes changes in cognitive processes such as attention, memory, ability to learn new skills, solve problems, reason, and make judgments, manipulation of symbols, language comprehension, perception, praxis and control over reactions and behaviors (Trivedi, 2006; Cotrena et al., 2017; Smirnova et al., 2017). Failures in these cognitive functions can result in inability to: pay attention, process quickly information, remembers and recall information, respond quickly to information, think critically, plan, organize and solve problems, for example (Trivedi, 2006). Then, it is evident that appropriate functioning of cognitive and emotional processes that enable people to deal with different types of social demands is imperative for living in a social context. In some countries, mental disorders are one of the principal reasons for granting social security disability benefits, and mood disorders are reported to be the most frequent (Silva-Junior and Fischer, 2015).

Several studies have investigated the cognitive performance of bipolar affective disorder (BD). The cognitive impairments reported whether in euphoria (mania/hypomania) and/or depression, involve reduced visuo-spatial skills, executive functions, processing speed, attentional process, working memory, and verbal memory (Osuji and Cullum, 2005; Volkert et al., 2016). Comparing the neuropsychological performance (attention, working memory, verbal memory, executive functioning) of 55 patients (35 depressed, 20 hypo-/manic) with 55 healthy controls, Volkert et al. (2016) found that depressed patients had psychomotor retardation as a characteristic, while manic patients had severe deficits in executive functioning. These authors found that in the euthymic phase, subclinical residual depressive symptoms had an impact on reduced processing speed, attention deficits and verbal memory. Other studies demonstrate cognitive impairment in making appropriate decisions and judgments (Osuji and Cullum, 2005; Cáceda et al., 2014; Volkert et al., 2016; Alexander et al., 2017; Ryu et al., 2017; Richa et al., 2018).

The literature has also emphasized the differences regarding reward sensitivity during episodes, that is, patients in a mood episode demonstrate difficulties in adjusting responses to intermittent rewards. Regarding the euthymic phase, there is no consensus on cognitive deficits (Bersani et al., 2016; Volkert et al., 2016; Ryu et al., 2017; Richa et al., 2018). In this setting, it appears that cognitive deficits are known as an underlying feature in BD; in addition, these deficits are associated with lower psychosocial functioning, affecting family, social, and professional lives (Sanchez-Moreno et al., 2009; Bennett et al., 2019; Sachs et al., 2020).

Although the literature emphasizes cognitive deficits in association with impaired psychosocial functioning in TB patients, few studies have investigated whether these deficits somehow impact a broader context of social life, such as legal capacity (that is, the ability to perform legal acts such as drawing up a will, selling a property, for example). Gergel et al. (2021) discussed the condition of treatment autonomy and the presence of conflicts regarding involuntary hospitalizations. Regarding the autonomy of treatment in BD patients, Gergel and Owen (2015) contextualized how feasible and legal pre-agreements to bind the treatment of these patients would be a way to deal with the mania phase of BD. However, the authors emphasize that this topic is complex and requires further study.

The current study aimed to verify the association between impairments in the cognitive process as attention, executive functioning, and legal capacity in patients with bipolar disorder. We hypothesized that episodic patients would express greater impairments in executive functioning compared to euthymic patients.

## Materials and Methods

### Participants and Procedures

**FIGURE 1:**
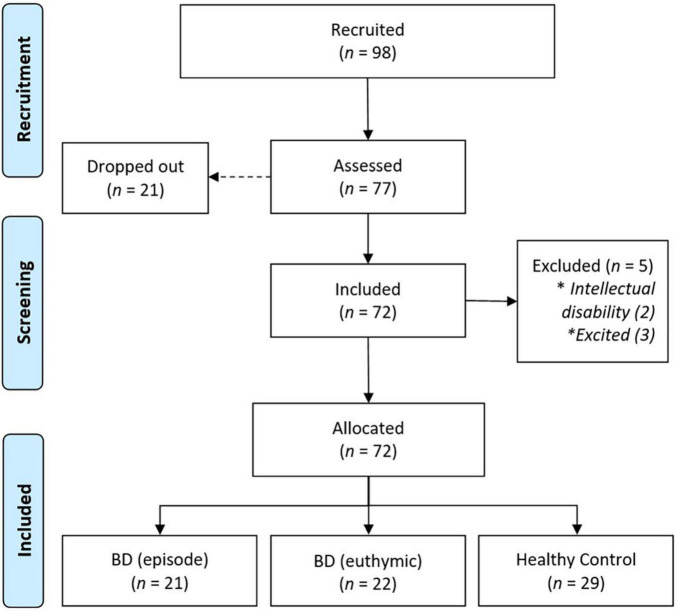
Flowdiagram of the selection and inclusion of the study.

Based on a power analysis for using G*Power 3.1.9.7 (Faul et al., 2007) *a priori*, with 80% power (Cohen, 1969), α = 0.05 and β = 0.4 (medium effects), we targeted a minimum sample size of 70 participants. This is a cross-sectional study that had initially enrolled 98 volunteers who were being treated in a hospital department. After the first call, 21 of these volunteers did not reappear, resulting in 77 participants. The exclusion criteria were as follows: (i) intellectual disability (*n* = 5, identified by applying the Wechsler Abbreviated Intelligence Scale—WASI); (ii) mania episode agitation condition (*n* = 3, these participants received medical advice not to perform activities that could be an additional stimulus); or (iii) psychotic symptoms. Seventy-two patients were included in the current study, allocated to three groups: (i) BD episodic (*n* = 21), mania/hypomania or depression; (ii) BD euthymic (*n* = 22); and healthy control (HC) (*n* = 29) without psychiatric history (Figure 1). The healthy control group had the same characteristics, except bipolar disorder. As the final sample is not enough to compare three clinical groups and a control, it was decided to join depressive and mania patient to just one group. This decision was based on a previous analysis by Independent Samples Test to consider the results of cognitive variables. There is no significant difference between them (depressive, *n* = 9, and mania, *n* = 11). Data were collected through the dissemination of the questionnaire on the social communication networks of the hospital.

The neuropsychological assessment protocol consisted of instruments for assessing the intellectual level and executive functioning. Data collection was carried out in a single meeting lasting approximately 90-min, in an individual modality, by neuropsychologists trained and specialized in mental health. Patients in episode were evaluated during psychiatric hospitalization. In Brazil, to use a neuropsychological instrument, it needs to be validated by Federal Council of Psychology (CFP). The choice of instruments was based on international publications on impaired cognitive functions in BD and considering the instruments validated by the CFP.

For the person to acquire full legal capacity, the executive function must be preserved, especially to the decision-making act. Part of this disability results from changes in cognitive processes such as attention, memory, language, and executive functions, which impact decision-making (Trivedi, 2006; Cotrena et al., 2017; Smirnova et al., 2017). Therefore, we assess these cognitive functions to discuss legal capacity.

The inclusion criteria were as follows: (i) being over 18 years old; (ii) having a BD diagnosis, euthymic or episodic (either in the depressive phase or in the manic/hypomanic phase); and (iii) having no previous psychiatric diagnosis and agreeing to participate in the study. BD patients were from a high-complexity public hospital.

### Measures

#### Conner’s Continuous Performance Test 3

The Conner’s continuous performance test 3 (CPT-3) is a computerized instrument used to assess attention and inhibitory control (Conners, 2018), described on the test as commissions: responding to stimuli that should not be responded to, it suggests impulsivity; perseverations: answers accomplished in less than 100 milliseconds after the presentation of a stimulus, indicating an anticipatory or impulsive response; detectability: the capacity for discriminating targets from non-targets; omissions: missing targets, in general, indicating absence of mind; and variability, which reflects the consistency measure of the response speed when comparing the subject with themselves, that is, how consistent is the response speed. In this version, the letters of the alphabet are presented one at a time for 250 ms, and the respondent is instructed to press the keyboard for each letter except the letter “X.” There are six blocks, each containing 3 subblocks of 20 letters each. The subblocks have different interstimulus intervals (ISIs) of 1, 2, or 4 s. This order varies between blocks. The letters are large, approximately 1 inch in size, and bold. The complete test takes approximately 14-min. Responses provide a measure of variance for response accuracy and reaction time (the greater the variance, the more inconsistent the participant’s attention) as well as traditional measures of omission’s errors, commission’s errors, and response times.

#### Wechsler Abbreviated Scale of Intelligence

The Wechsler abbreviated scale of intelligence (WASI) (Trentini et al., 2014) scale measures the intelligence of subjects between 6 and 89 years old, formed by four subtests: Vocabulary, Block Design, Similarities, and Matrix Reasoning. WASI vocabulary subtest reflects semantic knowledge and crystallized intelligence. Matrix reasoning reflects visual information processing and abstract/analog reasoning corresponding to fluid intelligence. Besides perceptual and visuospatial organizational skills, as well as visual analog reasoning. It takes 30-min to administer. An estimate of general intellectual ability may be obtained by using a form of two-subtests, including vocabulary and matrix reasoning. The two-subtest form takes approximately 15-min to administer. Subtests are scaled to a *T*-score metric.

#### Five Digit Test

The five digit test (FDT) (Sedó et al., 2015) measured the effect of attentional interference, inhibitory control, and mental flexibility. FDT is a numeric-Stroop paradigm applying four steps. Parts 1 and 2 involve automatic processes such as reading and counting. The Part 3 (choosing) involves interference control since an automatic numerical transcoding must be inhibited in favor of a controlled one. Part 4 (shifting) involves a set-shift from rules of Part 1 to Part 3 and vice-versa depending on an explicit marker. Executive scores are calculated for inhibition (choosing—reading) and flexibility (shifting—reading). The spent time is taken account to complete tasks in each part and errors are scored for each component of the test. The total test score is used as the main measure. Higher scores indicate worse performance.

#### Rey–Osterrieth Complex Figure

The Rey–Osterrieth complex figure (ROCF) (Rey, 2011) evaluated the visual-constructive ability, planning capacity, and problem-solving strategies. A measure of overall planning ability based on the order in which elements are drawn, their placement on the page and within the figure, and the overall integrity of the production. The arithmetic sum of the Fragmentation and Planning scores, providing an overall measure of organizational skills. ROCF performance accuracy is calculated by applying standard scoring criteria, which the geometry is divided into 18 units and scored on a 2-point scale for accuracy and placement. Thus, the maximum score for the copying task is 36. Higher scores indicate more efficient planning and a strategic approach to copy.

It also was used a sociodemographic questionnaire to collect the participants’ date of birth, age, education, sex, age at disease onset, number of psychiatric hospitalizations, comorbidities, and use of medications.

### Statistical Analysis

SPSS (Version 23.0 for Windows, IBM, Sao Paulo, SP, Brazil) was used for statistical analysis of the data. The homogeneity of the sample was verified using the Lèvene test. The Kolmogorov-Smirnov test was used to assess the normality of distribution of each quantitative variable studied. For homogeneous and Gaussian variables, one-way analysis of variance was used to test the differences between groups. In cases of significant *F*, Fisher’s Least Significant Difference *post hoc* test was performed for multiple comparisons. For non-homogeneous and/or non-Gaussian variables, the Kruskal–Wallis test was used in each group (considering comparisons of demographic, clinical and cognitive variables). In cases of significant *H*, Dunn’s *post hoc* test for multiple comparisons was performed. In the statistical analysis, *p* < 0.05 was considered to be statistically significant. The effect size of the statistically detected differences was obtained using the partial squared Eta index (η^2^_*p*_), which was used to calculate the effect size. Effect sizes of < 0.06, between 0.06 and 0.14, and > 0.14 were considered as small, medium, and large, respectively.

## Results

The sample was homogeneous in terms of age, duration of illness, number of psychiatric hospitalizations, and mood. Of the 21 episodic patients, 10 were in depression and 11 in mania. Most participants were female and this difference was significant in the euthymic group (Table 1).

**TABLE 1:** Characteristics of the participants.

	BD Episode^‡^(*n* = 21)	BD Euthymic (*n* = 22)	HC (*n* = 29)	*p*
			
	*Mdn* (IQR)	*Mdn* (IQR)	*Mdn* (IQR)	
Age, years	37 (31, 54)	39 (33, 46)	43 (38, 47)	0.89
Education, years^¶^	16 (12, 17)*	16 (13, 16)	17 (16, 20)	< 0.01
IQ^§^	93 (83, 104)*	107 (96, 113)	114 (104, 120)	< 0.01
Matrix reasoning	45 (37, 50)*	50 (44, 57)	54 (50, 59)	< 0.01
Vocabulary	45 (43, 64)*	54 (49, 59)	59 (54, 66)	0.01
Length of disease, years^¶^	11 (7, 18)	16 (8, 21)		0.58
Sex (F/M), %	57/43	91/9	76/24	0.04
Laterality (right-handed), %	97	96	95	0.83
Family antecedents, %	40	71	4	< 0.01
Suicide attempt, %	62	32	0	< 0.01
Psychotic symptoms, %	68	57		0.45
Inpatient				
Only once, %	33	24		0.5
2–5 times, %	57	33		0.12
Over 5 times, %	5	19		0.15

*Mdn, median; IQR, interquartile range (25th, 75th); HC, healthy control group; F/M, female/male.*

*^¶^ Kruskal–Wallis Test.*

*Difference vs. HC, p < 0.05.

*^‡^Mania/hypomania or depression.*

*^§^Intelligence quotient (IQ).*

In all groups, the average year of schooling corresponded to at least the completion of high school and/or beginning of college (8 or 9 years of elementary school plus 3 years of high school), but there was a significant difference between the episodic group and the HC group: the latter had more years of education. This same difference was noted with regard to intelligence quotient (IQ): for both fluid and crystallized intelligence, participants in the HC group showed significantly higher results than the episodic group, but not the euthymic group. Suicide attempt presented itself as a history exclusive to clinical groups, with greater frequency in the episodic group (*p* < 0.01). No differences were observed in the variables, psychotic symptoms, and number of hospitalizations (Table 2).

**TABLE 2:** Results of the groups for continuous performance test.

	BD Episode^‡^(*n* = 21)	BD Euthymic (*n* = 22)	HC (*n* = 29)	*p*	η^2^_*p*_
			
	*Mdn* (IQR)	*Mdn* (IQR)	*Mdn* (IQR)		
Commissions^¶^	49 (46, 57)*	50 (45, 61)*	41 (37, 46)	< 0.01	0.22
Perseverations^¶^	68 (48, 73)*^†^	47 (46, 48)	46 (46, 53)	< 0.01	0.19
Detectability	57 (51, 63)*^†^	54 (46, 60)*	43 (35, 48)	< 0.01	0.36
Omissions^¶^	56 (46, 76)*	48 (45, 53)	45 (44, 49)	< 0.01	0.15
Variability^¶^	58 (48, 69)*	53 (45, 57)	46 (42, 52)	< 0.01	0.13

*Mdn, median; IQR, interquartile range (25th, 75th); HC, healthy control group.*

*^¶^ Kruskal–Wallis Test.*

*Difference vs. HC, p < 0.05.

*^†^Difference vs. Euthymic group, p < 0.05.*

*^‡^Mania/hypomania or depression.*

While evaluating attention with CPT (Table 2), a statistical difference was noted between the episodic group and the HC group in the following aspects: commissions, perseverations, detectability, omissions, and variability (*p* < 0.01). In the comparison of the euthymic group with HC group, the euthymic group had the worst results in commissions (incorrect answer) and detectability (capacity for discriminating targets from not-targets) (*p* < 0.01). When comparing the patients in the clinical groups, there was a difference in perseverations (anticipatory, repetitive, or impulsive response) and detectability (ability to discriminate targets, non-targets) in the episodic group, indicating it would show the worst performance in these aspects (*p* < 0.01).

Table 3 shows the results of the assessment of executive functioning. Verified by the FDT, it is evident that patients in general (euthymic and episodic) are slower in their speed of processing compared to the HC group, both in automatic processes (which do not require extra effort) or in controlled processes (which require effort to maintain attention in front of distracting stimuli and in alternating between two operations). In addition, in the episodic phase, patients expressed less mental flexibility (they are more rigid), that is, they showed more difficulty in changing their attitude according to environmental demands. Thus, the results show that BD patients are slower in processing information when compared to the non-clinical population, and that in their episodic phase, they are more rigid with lower cognitive flexibility, not noticing or accepting environmental cues to change attitudes when necessary.

**TABLE 3:** Performance of the groups for five digit test and Rey–Osterrieth complex figure.

	BD Episode^‡^(*n* = 21)	BD Euthymic (*n* = 22)	HC (*n* = 29)	*p*	η^2^_*p*_
			
	*Mdn* (IQR)	*Mdn* (IQR)	*Mdn* (IQR)		
FDT—Time AP (seconds)^¶^	61 (45, 90)*	62 (52, 81)*	48 (42, 57)	< 0.01	0.14
FDT—Errors AP (raw score)^¶^	0 (0, 1)	0 (0, 1)	0 (0, 0)	0.05	
FDT—Time CP (seconds)^¶^	107 (89, 135)*	107 (92, 128)*	83 (68, 99)	< 0.01	0.16
FDT—Errors CP (raw score)	5 (1, 8)	3 (1, 8)	2 (1, 3)	0.05	
FDT—Inhibition (percentile)	45 (15, 71)	50 (15, 88)	55 (28, 83)	0.43	
FDT—Flexibility (percentile)	31 (6, 50)*	50 (25, 90)	60 (38, 90)	0.03	0.11
ROCF, copy (percentile)^¶^	40 (10, 100)*	73 (21, 100)	75 (75, 100)	0.03	0.07
ROCF, planning (raw score)^¶^	3 (1, 5)	5 (3, 6)		0.09	

*Mdn, median; IQR, interquartile range (25th, 75th); HC, healthy control group; FDT, Five Digit Test; AP, automatic process; CP, controlled process; ROCF, Rey–Osterrieth Complex Figure.*

*^¶^ Kruskal–Wallis Test.*

*Difference vs. HC, p < 0.05.

*^‡^Mania/hypomania or depression.*

With regard to the performance of visuospatial capacity, organizational skills, and action planning, it was observed that episodic BD patients would present lower performance in visuospatial organization when compared to HC group (Table 3). This result suggests that even if the planning capacity of BD patients is preserved, there is a loss in their orderliness or organization of actions, partly because they lose details that would help in responding to the environment appropriately.

## Discussion

In general, mental disorders are one of the factors that most contribute to the increase in disability worldwide (Okai et al., 2007; Merikangas et al., 2009; Millan et al., 2012). In Brazil, for example, mental disorders are the third main reason for granting social security benefits due to disability. Mood disorders have been the most frequent conditions (Silva-Junior and Fischer, 2015). The literature also emphasizes that cognitive deficit is a central feature in BD, with important repercussions for psychosocial functioning that also impacts the patients’ family, social, and professional lives (MacQueen et al., 2001; Dickerson et al., 2004; Sanchez-Moreno et al., 2009; Ryu et al., 2017; Richa et al., 2018; Bennett et al., 2019; Sachs et al., 2020). We carried out a study on BD patients to verify a possible association between impairments in the cognitive process such as attention, and executive functioning and discuss how these deficits might affect legal capacity, for example, the ability to perform legal acts such as a will, sale of property, among others.

In the general panorama of cognitive aspects, our results corroborate the results of existing studies. Compared to the HC group, BD patients in the episodic group had fewer years of schooling, even though their average was equivalent to that corresponding to the end of high school, corroborating both Brazilian data (Tucci et al., 2004; Ponsoni et al., 2021), and those from other countries (Torres et al., 2020). This suggested that BD presents itself as a condition that affects education in countries like Brazil.

Regarding IQ, no association was found between BD and intelligence impairments, once the results presented were in the mean range (IQ between 90 and 110). However, the clinical groups were different from the HC group, which obtained results in the upper mean range (IQ between 111 and 120). Furthermore, we noted a significant difference between the HC group and episodic group, but not between the HC group and euthymic group. These results have been corroborated by Vreeker et al. (2017) They affirmed that episodic BD patients tend to present lesser intellectual efficiency when compared to healthy subjects.

Furthermore, we observed that compared to the HC group, the episodic group participants responded more to stimuli that they should not have responded to, and hence, obtained lower results for discrimination and impulsive answers. Additionally, they presented lesser precision and inconsistency in response speed and more difficulty in organizing their actions. Thus, they showed more rigidity and lesser cognitive flexibility. These characteristics are related to executive functioning, which includes difficulty in controlling impulsiveness and, therefore, the action itself, as well as inattention and mental rigidity. When compared with euthymic patients, episodic patients had more anticipatory/repetitive/impulsive responses, with worse target discrimination, suggesting that episodic patients were more impulsive and less accurate. These characteristics tends to negatively compromise the patient proper understanding of situations and circumstances, which might lead to questioning within the scope of a legal process when their competence is questioned.

Euthymic patients, when compared to healthy individuals, had a slower speed of processing; they also made more errors in the analysis of the stimuli owing to the difficulty in discriminating what should or should not be done. This indicates that, in addition to being inattentive, they may also be impulsive to respond to a demand. Thus, considering the BD patients in episodic and euthymic groups, the study results suggest that while BD patients are slower in terms of analysis, they may also be more impulsive than the general population, their mood state being a determinant for impulsiveness. Regarding to suicide attempts and neurocognitive functioning, Fernández-Sevillano et al. (2021) studied cognition in patients that attempts to suicide and concluded that there were changes in the executive function performance when the episode was recent. However, in our sample, although 62% of patients in episode had been already suicide attempted, it was not recent.

In this context, our results converge with those reported in the literature, which has consistently described that BD patients have deficits in executive functioning, even in euthymia, with an emphasis on both lower response speed and tendencies for impulsivity (Osuji and Cullum, 2005; Tsitsipa and Fountoulakis, 2015; Bersani et al., 2016; Volkert et al., 2016; Alexander et al., 2017; Richa et al., 2018). In a study conducted by Bora et al. (2006), using the CPT-II, it was found that BD patients in mania make more errors (commissions) and have more perseveration, which indicated impulsivity. In their meta-analysis, Lewandowski et al. (2011) report deficits in executive functioning of BD patients in general, and add that as symptoms are more exacerbated, both for mania and depression, the deficits are more prominent.

Our results corroborate the findings in the existing literature, showing that the episodic group presented worse stimuli discrimination (detectability), and more errors (commissions) and omissions compared to the HC group. These skills are attentional aspects and involve working memory and processing speed (Osuji and Cullum, 2005; Volkert et al., 2016). In addition, the phenomenon of slowing down of processing speed in euthymic patients, when compared to healthy individuals, was also found in the study of Bora et al. (2006) and in a meta-analysis performed by Tsitsipa and Fountoulakis (2015).

It is important to consider that in a study conducted by Bora et al. (2006), using the CPT-II, they observed that BD patients in mania had inconsistency in response speed, as found in the present study with episodic BD patients. The authors argued that the inconsistency in response speed may even be an indicator of vulnerability in patients with BD. It is possible to consider even part of the attentional difficulty might be associated with BD comorbid for ADHD (Wingo and Ghaemi, 2007; Katzman et al., 2017).

Our results also showed that the episodic group had a lower performance in the visuospatial organization when compared to the HC group. This result indicates that, even though the planning capacity of episodic BD patients is preserved, their experience impairment in organizing their actions. In other words, for such individuals, inattention or even impulsiveness may lead to a loss of details in information processing, resulting in a decrease in the quality of actions, and therefore, may suggest that such difficulties need to be considered in terms of support (e.g., assisted decision making). Indeed, the impulsivity factor has been shown to be a variable present in BD patients (Bora et al., 2006; Lewandowski et al., 2011; Tsitsipa and Fountoulakis, 2015; Ramírez-Martín et al., 2020).

Therefore, it can be suggested that when the BD patients are in a mood episode, whether it is polarized to depression or to hypomania/mania, they present a cognitive deficit in attentional processes and working memory that might make it difficult to act in certain daily situations, including situations that involve important decisions in the judicial scope. Thus, it does not seem impractical to associate these deficits with the skills necessary for legal capacity. Considering the findings of this group in relation to inattention, impulsiveness, and difficulties in differentiating what is relevant from what is not fundamental for the moment, episodic BD patients may, for example, have difficulty in reading and properly understanding a contract document, miss important information in a negotiation, or sign a contract in an impulsive manner, not considering the possible long-term consequences, and aiming for only the immediate reward. Ryu et al. (2017) also discussed the difficulty of episodic BD patients to adjust their responses to intermittent rewards.

In the same manner, the difficulty in mental flexibility, that is, the rigidity presented by the members of the episodic group, might lead to difficulties in social interaction, as pointed out by Ponsoni et al. (2021) It might also, for example, lead to limited decision-making capacity and affect the understanding of more complex acts in the scope of legal capacity in a contractual negotiation.

In summary, the current study suggests that euthymic patients function slightly worse than healthy individuals and slightly better than episodic patients. A possible explanation for this could be the sub-syndromic symptoms in euthymic patients (Grunze and Born, 2020); however, these do not directly affect legal capacity, because other aspects, such as flexibility, order for acting in an environment, and fluid and crystallized intelligence, would be preserved. Nonetheless, regarding the performance of patients in a mood episode, the deficits observed in the current study will certainly lead to questions about their capacity for legal capacity in situations where the introduction of neuropsychological assessment would be configured as a necessary and protective expert resource. Peters et al. (2014) presented impairments in cognitive functions in bipolar patients for evaluating the patients with a scale that represents real-world settings and realized that mood symptom were the strongest predictor of subjective cognitive dysfunction, but they are not the only ones. Other factors like medicines, comorbidity, and illness chronicity also contribute to impairments in executive functions.

Previous studies have addressed impairments in cognitive functions, but, in general, they do not address legal capacity due to these impairments. Studies have discussed cognitive functions, as we have seen in a systematic review realized by Tsitsipa and Fountoulakis (2015) with 250 papers analyzed. Furthermore, our findings point out the need for bipolar patients, during episodes, to be supported in legal decisions, and it also may serve bipolar patients living in the community. Sanchez-Moreno et al. (2009), also indicated that impairments in cognitive functions affect bipolar patients’ quality of life even when they are in euthymia.

## Conclusion

In conclusion, the results of this study corroborate the findings of the existing literature. In addition, they produce relevant information, suggesting that BD patients in a mood episode (which is the period in which they are most vulnerable) have cognitive impairments. These impairments might negatively influence their functionality, progressing with a greater risk of impaired for decision-making, with implications for the legal capacity.

Another fact that deserves attention is that even in euthymic patients, there are cognitive functions decrease. Thus, although we have not evaluated it in this study, there is a need to discuss the role of decision-making in patients with mental disorders. Therefore, we have highlighted that to consider the relevance of cognitive deficits in the legal capacity of these patients, tools for practical assessment related to community functioning should be associated with the neuropsychological protocol.

In addition, the use of a more comprehensive neuropsychological battery with a measure of decision-making, would possibly produce more robust information and increase the generalization power of the results.

### Limitations

We also emphasize that in our sample, in relation to the clinical group, we observed the occurrence of suicide. The literature shows that this is a variable that corresponds to cognitive impairment, according to the severity of the attempt and the time elapsed (Fernández-Sevillano et al., 2021). As we did not control the type and possible sequelae, we emphasize that this is a feature limited to the present work, although in the Brazilian reality the legislation has a greater focus on considering the functional impact regardless of specificities such as, for example, a suicide attempt. In addition, we emphasize that although we have not carried out a systematic control of the medication used by the evaluated patients, which characterizes a limitation, we still consider the possible effects of this medication on the patient’s cognitive performance.

## Data Availability Statement

The original contributions presented in this study are included in the article/supplementary material, further inquiries can be directed to the corresponding author/s.

## Ethics Statement

The studies involving human participants were reviewed and approved by the Ethics Committee at the Clinics Hospital of the University of São Paulo Medical School—CAPPesq (approval number: 3.280.904) and was conducted in accordance with the principles of the National Health Council (Resolution no. 466/12), based on the Declaration of Helsinki. No participant received financial compensation for their assistance. The patients/participants provided their written informed consent to participate in this study.

## Author Contributions

FS, CR, and AS: study conception and design, data collection, and manuscript writing. ET-D and CR: data acquisition, data interpretation, and data analysis. FS and RD: drafting manuscript and figures. RD and AS: critical comments regarding the manuscript. All authors critically revised the article for intellectual content and approved the final version of the manuscript.

## Conflict of Interest

The authors declare that the research was conducted in the absence of any commercial or financial relationships that could be construed as a potential conflict of interest.

## Publisher’s Note

All claims expressed in this article are solely those of the authors and do not necessarily represent those of their affiliated organizations, or those of the publisher, the editors and the reviewers. Any product that may be evaluated in this article, or claim that may be made by its manufacturer, is not guaranteed or endorsed by the publisher.
